# Effects of Tai Chi Exercise on Balance Function in Stroke Patients: An Overview of Systematic Review

**DOI:** 10.1155/2022/3895514

**Published:** 2022-03-09

**Authors:** Caixia Hu, Xiaohui Qin, Minqing Jiang, Miaoqing Tan, Shuying Liu, Yuhua Lu, Changting Lin, Richun Ye

**Affiliations:** ^1^Department of Neurology, The Second Affiliated Hospital of Guangzhou University of Chinese Medicine (Guangdong Provincial Hospital of Chinese Medicine), Guangzhou, China; ^2^Guangzhou University of Chinese Medicine, Guangzhou, China

## Abstract

**Background:**

Tai chi (TC) has received increased attention in stroke rehabilitation, yet services are greatly underutilized. An increasing number of systematic reviews and meta-analyses (SRs/MAs) have begun to investigate the effects of TC on balance function in stroke patients. The aim of this current study was to systematically collate, appraise, and synthesize the results of these SRs/MAs using a systematic overview.

**Methods:**

Eight databases were searched: PubMed, Cochrane Library, Embase, Web of Science, CNKI, SinoMed, Chongqing VIP, and Wanfang Data. SRs/MAs of TC on balance function in stroke patients were included. Literature selection, data extraction, and assessment of the review quality were performed by two independent reviewers. Methodological quality was assessed by the Assessing the Methodological Quality of Systematic Reviews 2 (AMSTAR-2), reporting quality by Preferred Reporting Items for Systematic Reviews and Meta-Analyses (PRISMA), and evidence quality by Grading of Recommendations, Assessment, Development, and Evaluation (GRADE).

**Results:**

Nine SRs/MAs were included in this study. For methodological quality, what resulted in unsatisfactory methodological quality was noncompliance with critical item 4 (using a comprehensive literature search strategy) and critical item 7 (providing the list of excluded research literature). For reporting quality, what resulted in unsatisfactory reporting quality was inadequate reporting of Q1 (protocol and registration), Q8 (search), Q15 (risk of bias across studies), Q16 (additional analyses), Q22 (risk of bias across studies), Q23 (additional analysis), and Q27 (funding). For GRADE, the evidence quality was high in 0, moderate in 3, low in 11, and very low in 6. Risk of bias was the most common factor leading to downgrading of evidence, followed by inconsistency, imprecision, publication bias, and indirectness.

**Conclusions:**

TC may have beneficial effects on balance function in stroke survivors; however, this finding is limited by the generally low methodology, reporting quality, and evidence quality for published SRs/MAs.

## 1. Introduction

Stroke, a common and frequently occurring disease among elderly people, is considered to be the second most common cause of death and the third most common cause of disability worldwide [1].Stroke is a major global health challenge with a global incidence of 76-119 per 100,000 populations each year [2]. Despite the advances in stroke management in recent decades, up to 50% of stroke patients still have residual effects [3]. Impaired balance is the most common poststroke sequela [4] and an important predictor of functional independence after stroke [5]. Stroke survivors with impaired balance are more likely to fall and are associated with a higher risk of fracture, depression, anxiety, and even death [6]. Therefore, effective interventions to improve balance function in stroke survivors are urgently needed [7].

Various rehabilitation methods have been applied to improve balance functional training in stroke survivors [8], however with limited efficacy [9]. As a traditional martial art widely practiced in China for centuries, tai chi (TC) is well known for its slow and graceful rhythm transformation [10]. Recently, in December 2020, the United Nations Education Scientific and Cultural Organization (UNESCO) announced that TC has been included in the representative list of the intangible cultural heritage of humanity. TC was instilled by ancient Chinese philosophies and Chinese medicine, such as Confucian and Taoist cultures, and the ancient Chinese dialectical thinking can be reflected in the various movements of TC [11]. TC is characterized by fluidity and gentleness, calmness and consistency, and most importantly movement based on awareness [10]. As a mind-body exercise, TC involves the coordination of posture and breathing patterns, which are distinctly different from other aerobic exercises [12]. Performing TC exercises requires low space and equipment and almost zero cost, making it suitable for people of all ages in different conditions. There is ample evidence that tai chi is beneficial for dementia, depression, and cardiac and stroke rehabilitation [12]. For stroke rehabilitation, previous studies have shown that TC may be effective in improving balance, flexibility, and coordination and enhancing muscle strength, therefore helping to reduce the risk of falls [13]. Thus, TC has received increasing attention in stroke rehabilitation.

SRs/MAs are considered the gold standard for assessing the efficacy of clinical interventions, but the evidence derived from them is currently facing challenges due to the various risks of bias generated during the formation of evidence by SRs/MAs [14]. High-quality SRs/MAs can provide reliable evidence, while low-quality SRs/MAs may instead mislead decision-makers [15]. Thus, the evidence of uneven quality leads to a gap between its use and practical implementation in real-world dynamics. Where multiple SRs/MAs are published for overlapping topics in a relatively short time frame, an overview is needed to systematically collate, evaluate, and synthesize the evidence from these SRs/MAs [16]. The ultimate goal of an overview is to provide a comprehensive evaluation of the current evidence on multiple identical topics, to provide more focused high-quality evidence to evidence users, and to identify key flaws in evidence use [14]. A literature search yielded a growing number of systematic reviews, and the meta-analyses (SRs/MAs) have examined the effects of TC on balance function of stroke patients. To systematically collate, appraise, and synthesize the results of these SRs/MAs, we carried out this study.

## 2. Methods

This study was conducted according to the Cochrane Handbook [17] and some high-quality methodological articles [10, 11]. The protocol was prospectively registered on the PROSPERO (CRD42021240693).

### 2.1. Inclusion and Exclusion Criteria

#### 2.1.1. Type of Studies

SRs/MAs only included clinical random control trails (RCTs) investigating the therapeutic effects of TC on balance function in stroke patient. No language constraints were placed on this study.

#### 2.1.2. Types of Subjects

The subjects were diagnosed as stroke, regardless of age, sex, or race.

#### 2.1.3. Types of Interventions

The experimental intervention was the use of TC in stroke survivors with conventional rehabilitation therapy (CRT) as the control intervention.

#### 2.1.4. Types of Outcomes

The outcome indicators focused on balance and gait after stroke, e.g., short physical performance battery (SPPB), functional reach test (FRT), dynamic gait index (DGI), timed up-and-go test (TUG), Holden walking grading scale, Berg balance scale (BBS), Fugl-Meyer assessment scale (FMA), and fall rates.

### 2.2. Search Strategy

PubMed, Cochrane Library, Embase, Web of science, CNKI, Chongqing VIP, SinoMed, and Wanfang Data were searched from their inception to April 2021. The following keywords were used: Tai Chi, stroke, balance, systematic review, and meta-analysis. Detailed search strategy in PubMed was given in Appendix file 1.

### 2.3. Eligibility Assessment and Data Extraction

Search results were imported into Endnote. The titles and abstracts were screened by two independent reviewers firstly; the potential full texts were then evaluated to determine the final eligibility. Any discrepancies were solved by introducing a third researcher for judgment.

Data were extracted from each included SRs/MAs using a predefined form by two independent reviewers. Information of authors, published year, country, sample, quality assessment tool, interventions, comparisons, outcome measures, data synthesis methods, and main results were extracted. Any discrepancies were solved by introducing a third researcher for judgment.

### 2.4. Review Quality Assessment

The methodological quality, reporting quality, and evidence quality were assessed by two independent reviewers using the Assessing the Methodological Quality of Systematic Reviews 2 (AMSTAR-2) tool [18] and the Preferred Reporting Items for Systematic Reviews and Meta-Analyses (PRISMA) [19], respectively. Any discrepancies were solved by introducing a third researcher for judgment. For evidence quality, each meta-analysis for the outcome of interest was assessed by two independent reviewers using the Grading of Recommendations Assessment, Development and Evaluation (GRADE) system [20]. Any discrepancies were solved by introducing a third researcher for judgment. Detailed items for AMSTAR-2 are provided in Additional file 2 and for PRISMA in Additional file 3.

## 3. Results

### 3.1. Results on Literature Selection

**Figure 1:**
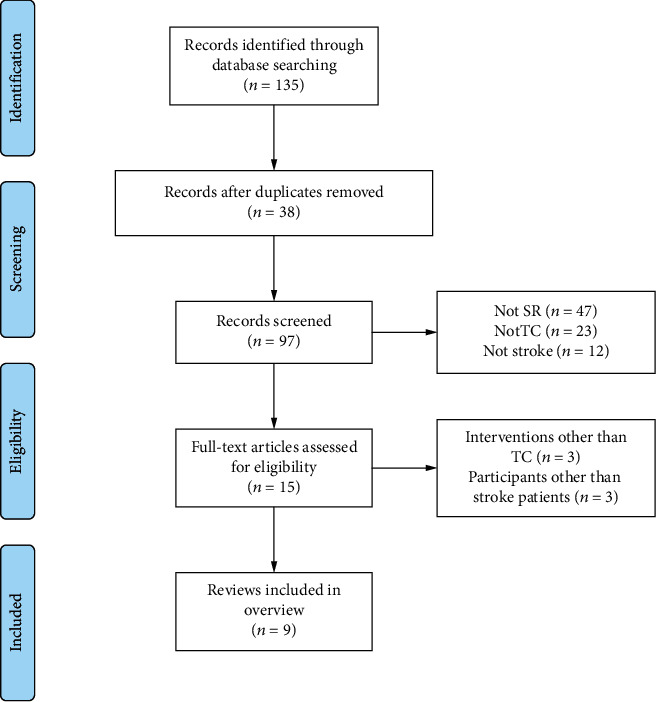
Literature selection procedure.

The electronic searches resulted in 135 articles. After removal of duplicates, 82 were excluded at the title and abstract stage. Fifteen articles were retrieved for examination on full text, with 9 reviews [21–29] finally included.  Figure 1 outlines the process of identifying the qualified articles.

### 3.2. Studies Characteristics

All studies were published between 2016 and 2021, with 5 [21–25] written in English and 4 in Chinese [26–29]. Authors of all included reviews were from China. The number of RCTs within each review ranged from 7 to 21, and the participants in these RCTs ranged from 346 to 1297. TC or TC plus CRT was used in the treatment group, and CRT was used lonely in the control group. All reviews evaluated the risk of bias of the RCTs, 3 used the Jadad scale, and the remaining 6 used Cochrane risk of bias criteria. All reviews conducted a meta-analysis, and both of them reached positive results. Details are presented in Table 1.

### 3.3. Quality Assessment

#### 3.3.1. Methodological Quality

**Figure 2:**
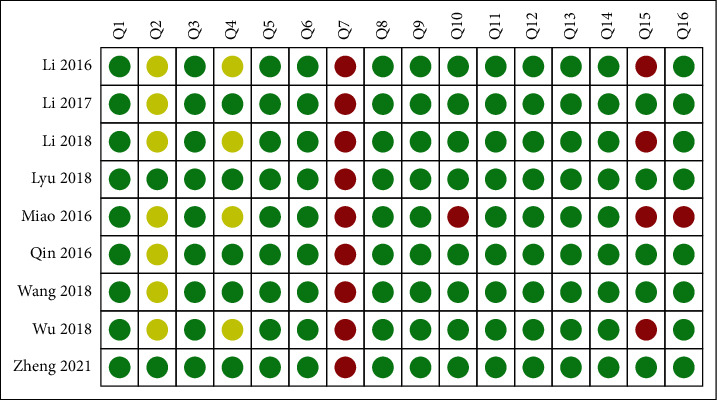
AMSTAR-2 assessments.

The results of methodological quality are reported in Figure 2. The main weakness affecting the methodological quality was the following: only 2 (22.2%) reviews registered a protocol before conducting the study; no (0%) reviews provided the list of excluded research literature; only 5 (55.6%) reviews provided the use of a specific search strategy; only 6 (66.7%) reviews carried out an investigation of publication bias and discussed its likely impact on the results; and 11.1% did not report the funding source and declare the conflicts of interest. Based on the above assessment, all reviews were judged to provide “low/critically low” methodological quality.

#### 3.3.2. Reporting Quality

The results of reporting quality are reported in Table 2. In summary, only two studies reported all items of PRISMA, and the reaming studies were reported over 75%. The main weakness affecting the reporting quality was the following: only22.2% reviews reported the topic of the protocol and registration, 55.6% reported specific search strategy, 77.8% reported risk of bias across studies, and 66.7% reported additional analyses in the section of the methods; for the result section, 88.9% reported the risk of bias, and 66.7% reported additional analyses; for the funding section, it was reported in only 88.9% reviews.

#### 3.3.3. Evidence Quality

The results of evidence quality are reported in Table 3.Twenty outcomes related to the effectiveness of TC on balance function of stroke patients were included. Among these outcome indicators, the evidence quality was high in 0 (0/20.0%), moderate in 3 (3/20.15%), low in 11 (11/20.55%), and very low in 6 (6/20.30%).

### 3.4. Efficacy Evaluation

Narrative synthesis was performed for all included outcomes. When the TC group was compared with controls, there were a significant effect for better BBC in 7 reviews; a significant effect for better FMA, balance, and walking in 2 reviews; and a significant effect for TUG, Holden scores, and gait ability in 1 review. However, there were no significant difference in SPBB and FMA between the TC and controls in 2 reviews and no significant difference in balance and walking in 1 reviews. More details are presented in Table 3.

## 4. Discussion

Stroke rehabilitation has always been a key health concern worldwide. Complementary and alternative therapies hold promise for improving the quality of life of stroke survivors [30]. TC is widely used in China for functional recovery exercises after stroke, and bibliometric evidence supports that TC may be a frontiers and promising field for stroke rehabilitation [31]. The core of the overview is a comprehensive evaluation of current SR/MA evidence on multiple identical topics, providing a more focused evidence base for users of the evidence [32]. A literature search yielded a growing number of SRs/MAs that examined the effects of TC on balance function of stroke patients. However, the quality of these SRs/MAs has not been evaluated, and their results were not completely consistent. Hence, to systematically collate, appraise, and synthesize the results of these SRs/MAs, a systematic overview was conducted.

### 4.1. Summary of Main Findings

First, this overview identified 9SRs/MAs which contained evidence relevant to the effects of TC on balance function of stroke patients. However, the methodological quality, reporting quality, and evidence quality of the included SRs/MAs were unsatisfactory. For methodological quality, the evaluation results of AMSTAR-2 showed that all included SRs/MAs had one or more critical items that were unmet; thus, these SRs/MAs were all judged to be of low or critically low methodological quality. What resulted in unsatisfactory methodological quality was noncompliance with critical item 4 (using a comprehensive literature search strategy) and critical item 7 (providing the list of excluded research literature). For reporting quality, the evaluation results of PRISMA showed that only 2 reviews had reported all items, and the reaming reviews had various degrees of missing report content. What resulted in unsatisfactory reporting quality was inadequate reporting of Q1 (protocol and registration), Q8 (search), Q15 (risk of bias across studies), Q16 (additional analyses), Q22 (risk of bias across studies), Q23 (additional analysis), and Q 27 (funding). For evidence quality, twenty outcomes were included; however, the evidence quality was high in 0 and moderate in 6, and the reaming were all judged to be of low or critically low evidence quality. Risk of bias was the most common factor leading to downgrading of evidence, followed by inconsistency, imprecision, publication bias, and indirectness.

Second, definitive conclusions of the effects of TC on balance function of stroke patients cannot be drawn based on the published SRs/MAs. In this overview, all included SRs/MAs concluded positive finding of the effects of TC on balance function in stroke patients. However, most authors of these SRs/MAs did not want to draw firm conclusions due to the small size of the included RCTs or their low quality. Furthermore, as we all know, only SRs/MAs with high quality will be helpful to provide scientific evidence. However, the evaluation results of this overview found that the overall methodological quality, reporting quality, and evidence quality of the included SRs/MAs were unsatisfactory, indicating that there were limitations in the reliability of the conclusions of these included SRs/MAs. Hence, caution should be warranted when recommending TC for the rehabilitation of stroke patients.

Third, the authors of these included SRs/MAs used the term “tai chi” to represent all types of this exercise. Actually, TC was performed in a variety of forms, which were named after different Chinese families such as Chen, Yang, Wu, and Sun. Different types obviously can have inconsistent effects; thus, all types of TC will be represented by one term in the included SRs/MAs that could lead to a source of heterogeneity. The evaluation results of the evidence quality in this overview revealed that inconsistency was one of the important factors for evidence degradation, which further verified that it may be unreasonable to represent this exercise in the term of “tai chi” and that subgroup analysis for different types of TC in SRs/MAs is still necessary. In addition, the variety of TC protocols, including differences in training style, form, frequency, and duration, may have contributed to the source of heterogeneity in the included SRs/MAs. Recommendations regarding TC parameters need to be standardized.

### 4.2. Application, Mechanism, and Frontier of TC in Stroke Rehabilitation

TC has been used in stroke rehabilitation worldwide for more than 10 years [33]. In recent years, researchers have increasingly focused on the role of TC in improving balance function in stroke survivors [21–29]. The improvement of balance function by TC is part of the comprehensive rehabilitation of stroke survivors and has an inherent relationship with functional rehabilitation. Balance function is closely associated with muscle strength, especially in the lower limbs. A recent 3D kinetic study reported that TC leads to similar mechanical behavior of biologically based tissues that can enhance lower limb strength, which in turn can help improve balance function and prevent falls [34]. Another electromyography study found similar results of increased lower limb muscle strength and improved neuromuscular responses after practicing TC for one year [35]. In addition, for stroke survivors with lower extremity paralysis, wheelchair TC can also help improve upper extremity mobility [36]. In terms of mechanistic studies, TC has been reported to act by modulating the neural function and biomechanics of balance; that is, TC improves neuromuscular responses and enhances balance function by controlling the stepping strategy of the swinging leg [37, 38]. In recent years, functional MRI and electroencephalography have been used to study central mechanisms. It was found that bilateral dorsolateral prefrontal cortex and hippocampus with enhanced functional connectivity and low frequency fluctuations in amplitude were observed in TC-trained patients [39]. In addition, meaningful functional and structural changes in the default mode network were detected, suggesting that tai chi helps improve patients' attention, which in turn affects balance and walking ability [40]. Greater amplitude of P3b event-related potential switching trials was observed from electroencephalography, suggesting that TC may promote peripheral nervous system recovery through central action [41].

In addition, TC also contributes to the recovery of other functions after stroke, such as cognitive function [42]. The mechanism lies in the positive impact on the patient's immune system by promoting DNA repair and lymphocyte renewal [43].

### 4.3. Implications for Practice and Research

The evaluation results identified common areas for improvement. First, study protocols should be registered in advance, which is essential to ensure the rigor of the SR/MA and to avoid any possible risk of bias. Second, specific and used search strategies for the exclusion of literature lists should be provided to ensure reproducibility of studies, improve transparency, and avoid publication bias. Third, the scientific nature of the analytical methods should be considered when conducting data analysis. For example, the subgroup analysis may be performed to address study heterogeneity. In addition, because commercially funded research yields results that may be biased in favor of the funder, the source of funding and any conflicts of interest should be fully reported. In summary, the quality of currently published SR/MA is unsatisfactory, and the defects that lead to the low quality have been clearly shown in this overview; future researchers should carry out the SRs/MAs in strict accordance with the standards to ensure the provision of high-quality evidence.

### 4.4. Limitations

This study uses the method of overview to systematically collate, appraise, and synthesize the results of SRs/MAs regarding to the effects of TC on balance function in stroke patients. However, limitations need to be acknowledged. First, it can be expected that there will be some overlapping trials in the included SRs/MAs. However, these overlaps have not been systematically explored, which may lead to double counting of data in the reported meta-analysis. Second, SRs/MAs included in this overview used the term “tai chi” to represent all types of this exercise, and relevant information on the different types of TC was lacking, so this overview cannot draw recommendations for the use of specific types of TC. Additionally, in the context of the coronavirus disease 2019 (COVID-19) pandemic, the isolation may lead to less exercise and more health-related problems; hence, the effects of TC on balance function and stroke patients under COVID were not included in is a pity.

## 5. Conclusion

TC may have beneficial effects on balance function in stroke survivors; however, this finding is limited by the generally low methodology, reporting quality, and evidence quality for published SRs/MAs.

## Figures and Tables

**Table 1:** Study characteristics.

Reviews	Country	Simple	Treatment intervention	Control intervention	Quality assessment	Meta-analysis	Conclusions
Zheng [21]	China	8 (1297)	TC and TC+CRT	CRT	Cochrane criteria	Yes	Balance functions and exercise capacities of stroke patients improved after they did TC exercise regularly.

Wu [22]	China	6 (347)	TC and TC+CRT	CRT	Jadad	Yes	These findings indicated that TC is superior to the CRT in the improvement of balance function, gait speed, and quality of life.

Li [23]	China	5 (346)	TC and TC+CRT	CRT	Cochrane criteria	Yes	TC may be beneficial for balance function in stroke survivors in the short term, but further RCTs with large sample sizes and long-term follow-up are needed to confirm this conclusion.

Lyu [24]	China	21 (1293)	TC and TC+CRT	CRT	Cochrane criteria	Yes	TC was beneficial on ADL, balance, limb motor function, and walking ability among stroke survivors.

Li [25]	China	17 (1209)	TC	CRT	Cochrane criteria	Yes	TC was superior to the CRT in the improvement of balance function and quality of life. However, there were no significant differences in walking function.

Wang [26]	China	8 (408)	TC and TC+CRT	CRT	Jadad	Yes	TC was superior to the CRT in the improvement of balance ability and motor function.

Qin [27]	China	15 (1016)	TC	CRT	Jadad	Yes	These findings indicated that TC was superior to the CRT in the improvement of balance function, gait speed, and quality of life.

Miao [28]	China	9 (698)	TC and TC+CRT	CRT	Cochrane criteria	Yes	The study indicated that TC could improve the balance function for stroke patients. However, further large, long-term RCTs with standard evaluation indicators are needed to confirm this conclusion.

Li [29]	China	7 (629)	TC and TC+CRT	CRT	Cochrane criteria	Yes	These findings indicated that TC was superior to the CRT in the improvement of balance function, gait speed, and quality of life.

**Table 2:** Result of the PRISMA assessments.

Section/topic	Items	Zheng 2021	Wu 2018	Li 2018	Lyu 2018	Li 2017	Wang 2018	Qin 2016	Miao 2016	Li 2016	Compliance (%)
Title	Q1	Y	Y	Y	Y	Y	Y	Y	Y	Y	100%
Abstract	Q2	Y	Y	Y	Y	Y	Y	Y	Y	Y	100%
Introduction	Q3	Y	Y	Y	Y	Y	Y	Y	Y	Y	100%
	Q4	Y	Y	Y	Y	Y	Y	Y	Y	Y	100%
Methods	Q5	Y	N	N	Y	N	N	N	N	N	22.2%
	Q6	Y	Y	Y	Y	Y	Y	Y	Y	Y	100%
	Q7	Y	Y	Y	Y	Y	Y	Y	Y	Y	100%
	Q8	Y	PY	PY	Y	Y	Y	Y	PY	PY	55.6%
	Q9	Y	Y	Y	Y	Y	Y	Y	Y	Y	100%
	Q10	Y	Y	Y	Y	Y	Y	Y	Y	Y	100%
	Q11	Y	Y	Y	Y	Y	Y	Y	Y	Y	100%
	Q12	Y	Y	Y	Y	Y	Y	Y	Y	Y	100%
	Q13	Y	Y	Y	Y	Y	Y	Y	Y	Y	100%
	Q14	Y	Y	Y	Y	Y	Y	Y	Y	Y	100%
	Q15	Y	Y	N	Y	Y	Y	Y	Y	N	77.8%
	Q16	Y	N	N	Y	Y	Y	Y	N	Y	66.7%
Results	Q17	Y	Y	Y	Y	Y	Y	Y	Y	Y	100%
	Q18	Y	Y	Y	Y	Y	Y	Y	Y	Y	100%
	Q19	Y	Y	Y	Y	Y	Y	Y	Y	Y	100%
	Q20	Y	Y	Y	Y	Y	Y	Y	Y	Y	100%
	Q21	Y	Y	Y	Y	Y	Y	Y	Y	Y	100%
	Q22	Y	Y	N	Y	Y	Y	Y	Y	Y	88.9%
	Q23	Y	N	N	Y	Y	Y	Y	N	Y	66.7%
Discussion	Q24	Y	Y	Y	Y	Y	Y	Y	Y	Y	100%
	Q25	Y	Y	Y	Y	Y	Y	Y	Y	Y	100%
	Q26	Y	Y	Y	Y	Y	Y	Y	Y	Y	100%
Funding	Q27	Y	Y	Y	Y	Y	Y	Y	N	Y	88.9%

**Table 3:** Results of evidence quality.

Review	Outcomes	Certainty assessment							No. of patients		Relative effect (95% CI)	*P* value	Quality
		No. of trails	Design	Limitations	Inconsistency	Indirectness	Imprecision	Publication bias	Experimental	Control			
Zheng [21]	BBC	6	Rct	Serious^a^	Serious^b^	No	No	No	231	181	MD 7.67 (3.44, 11.90)	<0.001	⨁⨁⨁◯◯ Low
	FMA	5	Rct	Serious^a^	Serious^b^	No	No	No	335	321	MD 4.15 (1.68, 6.63)	0.001	⨁⨁⨁◯◯ Low
	SPPB	2	Rct	Serious^a^	No	No	Serious^c^	No	69	60	MD -0.22 (-1.00, 0.56)	0.589	⨁⨁⨁◯◯ Low

Wu [22]	BBC	3	Rct	Serious^a^	Serious^b^	No	Serious^c^	Serious^d^			MD 4.823 (2.138, 7.508)	<0.001	⨁◯◯◯◯ Very low
	SPPB	2	Rct	Serious^a^	Serious^b^	No	Serious^c^	Serious^d^			MD 0.293 (-0.099, 0.685)	0.14	⨁◯◯◯◯ Very low

Li [23]	Gait ability (TUG and SPBB)	4	Rct	Serious^a^	No	No	No	Serious^d^	151	133	SMD -0.26 (-0.50, -0.03)	0.027	⨁⨁⨁◯◯ Low
	Balance (SPBB, DGI, and FRT)	3	Rct	Serious^a^	No	No	No	Serious^d^	77	71	SMD 0.15 (-0.27, 0.58)	0.475	⨁⨁⨁◯◯ Low

Lyu [24]	FMA	3	Rct	Serious^a^	Serious^b^	No	Serious^c^	No	85	81	MD 2.75 (0.95, 4.56)	0.003	⨁⨁◯◯◯ Very low
	BBS	2	Rct	Serious^a^	No	No	Serious^c^	No	75	75	MD 5.23 (3.42, 7.05)	<0.001	⨁⨁⨁◯◯ Low
	Holden scale	3	Rct	Serious^a^	No	No	Serious^c^	No	94	92	MD 0.61 (0.38, 0.85)	<0.001	⨁⨁⨁◯◯ Low
	TUG	5	Rct	Serious^a^	No	No	No	No	200	180	MD 2.59 (1.76, 3.43)	<0.001	⨁⨁⨁⨁◯ Moderate

Li [25]	BBS	9	Rct	No	Serious^b^	No	No	No	333	337	MD 9.34 (6.49, 12.19)	<0.001	⨁⨁⨁⨁◯ Moderate
	Walking (TUG and Holden scale)	4	Rct	No	Serious^b^	No	No	No	259	248	MD 0.84 (-0.31, 0.55)	0.05	⨁⨁⨁⨁◯ Moderate

Wang [26]	BBS	6	Rct	Serious^a^	Serious^b^	No	Serious^c^	No	118	118	SMD 2.49 (0.90, 4.07)	<0.001	⨁⨁◯◯◯ Very low
	FMA	3	Rct	Seriousa	Seriousb	No	Serious^c^	No	64	64	SMD 0.84 (-0.91, 2.58)	0.35	⨁⨁◯◯◯ Very low

Qin [27]	Balance (BBS, SPBB, and DGI)	9	Rct	Serious^a^	Serious^b^	No	No	No	283	275	MD 2.49 (0.90, 4.07)	<0.001	⨁⨁⨁◯◯ Low
	Walking (SPBB and TUG)	4	Rct	Seriousa	Seriousb	No	No	No	129	120	MD 0.27 (0.04, 0.50)	0.02	⨁⨁⨁◯◯ Low

Miao [28]	BBS	7	Rct	No	Serious^b^	No	No	Serious^d^	344	379	MD 11.43 (7.43, 15.42)	<0.001	⨁⨁⨁◯◯ Low
	FMA	2	Rct	No	Serious^b^	No	Serious^c^	Serious^d^	110	114	MD 12.77 (-5.07, 30.60)	0.16	⨁⨁◯◯◯ Very low

Li [29]	BBS	2	Rct	Serious^a^	No	No	No	Serious^d^	283	306	MD 6.36 (5.23, 7.49)	<0.01	⨁⨁⨁◯◯ Low

CI: confidence interval; MD: mean difference; SMD: standardized mean difference; ^a^the experimental design had a large bias in random and distributive findings or was blind; ^b^the confidence interval overlap less, the heterogeneity test *P* was very small, and the *I*^2^ was larger; ^c^the confidence interval was not narrow enough, or the simple size is too small; ^d^funnel graph asymmetry, or fewer studies were included, and there may have been greater publication bias.

## Data Availability

All analyses were based on previously published studies; thus, no informed consent is required.

## Abbreviations

TC: Tai chi
SR: Systematic review
MA: Meta-analysis
AMSTAR-2: Assessing the Methodological Quality of Systematic Reviews 2
PRISMA: Preferred Reporting Items for Systematic Reviews and Meta-Analyses
GRADE: Grading of Recommendations, Assessment, Development, and Evaluation
RCT: Random control trails
CRT: Conventional rehabilitation therapy
BBS: Berg balance scale
SPBB: Short physical performance battery
FRT: Functional reach test
FGI: Dynamic gait index
TUG: Timed up-and-go test
FMA: Fugl-Meyer assessment.

## Supplementary Materials

Supplementary Materials Appendix 1: search strategies of PubMed. Additional file 2: the 16 items of AMSTAR-2. Additional file 3: the 27 checklists of PRISMA.
