# Supplementary material for: Effects of Tai Chi Exercise on Balance Function in Stroke Patients: An Overview of Systematic Review
Source: Neural Plast. 2022 Mar 9;2022:3895514. doi: 10.1155/2022/3895514 (PMC8926482; doi:10.1155/2022/3895514)
Supplement: Supplementary Materials — Appendix 1: search strategies of PubMed. Additional file 2: the 16 items of AMSTAR-2. Additional file 3: the 27 checklists of PRISMA. [file 3895514.f1.pdf]

Appendix 1. Search strategies of Pubmed

#1 Cerebral Hemorrhage[Mesh] OR Brain Infarction[Mesh] OR Stroke[Mesh] OR Cerebrovascular Disorders[Mesh]

#2 Cerebro-vascular Disorder*[Title/Abstract] OR Intracranial Vascular Disease*[Title/Abstract] OR Intracranial Vascular Disorder*[Title/Abstract] OR Cerebro-vascular Disease*[Title/Abstract] OR Brain Vascular Disorder*[Title/Abstract] OR Cerebro-vascular Occlusion*[Title/Abstract] OR Cerebro-vascular Insufficiency[Title/Abstract] OR Cerebro-vascular Insufficiencies OR Stroke*[Title/Abstract] OR Cerebro-vascular Accident*[Title/Abstract] OR CVA*[Title/Abstract] OR Cerebro-vascular Apoplexy[Title/Abstract] OR Brain Vascular Accident*[Title/Abstract] OR Cerebro-vascular Stroke*[Title/Abstract] OR Apoplexy[Title/Abstract] OR Cerebral Stroke*[Title/Abstract] OR Acute Stroke*[Title/Abstract] OR Acute Cerebro-vascular Accident*[Title/Abstract] OR Brain Infarction*[Title/Abstract] OR Brain Infarct*[Title/Abstract] OR Anterior Circulation Brain Infarction*[Title/Abstract] OR Brain Venous Infarction*[Title/Abstract] OR Anterior Cerebral Circulation Infarction*[Title/Abstract] OR Posterior Circulation Brain Infarction*[Title/Abstract] OR Cerebrum Haemorrhag*[Title/Abstract] OR Cerebral Parenchymal Haemorrhag*[Title/Abstract] OR Intracerebral Haemorrhag*[Title/Abstract] OR Cerebral Haemorrhag*[Title/Abstract] OR Cerebral Brain Haemorrhag*[Title/Abstract]

#3 #1 OR #2

#4 Balance[Title/Abstract] OR static balance[Title/Abstract] OR dynamic balance[Title/Abstract] OR postural control[Title/Abstract] OR stability limit[Title/Abstract] OR equilibrium[Title/Abstract] OR postural stability [Title/Abstract]

#5 Tai ji[Mesh]

#6 Tai ji[Title/Abstract] OR Tai-ji[Title/Abstract] OR Tai Chi[Title/Abstract] OR Tai Ji Quan[Title/Abstract] OR Taiji[Title/Abstract] OR Taijiquan[Title/Abstract] OR Tai Chi Chuan[Title/Abstract]

#7 #5 OR #6

#8 Systematic Review [Publication Type] OR Meta-Analysis [Publication Type]

#9 Meta-Analysis as Topic[Mesh] OR Systematic Reviews as Topic[Mesh]

#10 Systematic Review as Topic[Title/Abstract] OR Systematic reviews as topic[Title/Abstract] OR Systematic Review*[Title/Abstract] OR Cochrane Review*[Title/Abstract] OR Systematic Evaluation*[Title/Abstract] OR Systematic Assessment*[Title/Abstract] OR Meta-Analysis as Topic[Title/Abstract] OR Meta Analysis as Topic[Title/Abstract] OR Meta-analytic*[Title/Abstract] OR Meta-analyses[Title/Abstract] OR Metaanalysis[Title/Abstract] OR Meta Analysis[Title/Abstract] OR Data Pooling*[Title/Abstract] OR Clinical Trial Overview*[Title/Abstract]

#11 #8 OR #9 OR #10

#14 #3 AND #4 AND #7 AND #11

Additional file 2: The 16 items of AMSTAR-2

Q1: Did the research questions and inclusion criteria for the review include the components of PICO?
Q2: Did the report of the review contain an explicit statement that the review methods were established prior to the conduct of the review and did the report justify any significant deviations from the protocol?
Q3: Did the review authors explain their selection of the study designs for inclusion in the review?
Q4: Did the review authors use a comprehensive literature search strategy?
Q5: Did the review authors perform study selection in duplicate?
Q6: Did the review authors perform data extraction in duplicate?
Q7: Did the review authors provide a list of excluded studies and justify the exclusions?
Q8: Did the review authors describe the included studies in adequate detail?
Q9: Did the review authors use a satisfactory technique for assessing the risk of bias (RoB) in individual studies that were included in the review?
Q10: Did the review authors report on the sources of funding for the studies included in the review?
Q11: If meta-analysis was performed did the review authors use appropriate methods for statistical combination of results?
Q12: If meta-analysis was performed, did the review authors assess the potential impact of RoB in individual studies on the results of the meta-analysis or other evidence synthesis?
Q13: Did the review authors account for RoB in individual studies when interpreting/discussing the results of the review?
Q14: Did the review authors provide a satisfactory explanation for, and discussion of, any heterogeneity observed in the results of the review?
Q15: If they performed quantitative synthesis, did the review authors carry out an

adequate investigation of publication bias (small study bias) and discuss its likely impact on the results of the review?

Q16: Did the review authors report any potential sources of conflict of interest, including any funding they received for conducting the review.

Additional file 3: The 27 checklists of PRISMA

Section/topic	#	Checklist item
**TITLE**		
Title	1	Identify the report as a systematic review, meta-analysis, or both.
**ABSTRACT**		
Structured summary	2	Provide a structured summary including, as applicable: background; objectives; data sources; study eligibility criteria, participants, and interventions; study appraisal and synthesis methods; results; limitations; conclusions and implications of key findings; systematic review registration number.
**INTRODUCTION**		
Rationale	3	Describe the rationale for the review in the context of what is already known.
Objectives	4	Provide an explicit statement of questions being addressed with reference to participants, interventions, comparisons, outcomes, and study design (PICOS).
**METHODS**		
Protocol and registration	5	Indicate if a review protocol exists, if and where it can be accessed (e.g., Web address), and, if available, provide registration information including registration number.
Eligibility criteria	6	Specify study characteristics (e.g., PICOS, length of follow-up) and report characteristics (e.g., years considered, language, publication status) used as criteria for eligibility, giving rationale.
Information sources	7	Describe all information sources (e.g., databases with dates of coverage, contact with study authors to identify additional studies) in the search and date last searched.
Search	8	Present full electronic search strategy for at least one database, including any limits used, such that it could be repeated.
Study selection	9	State the process for selecting studies (i.e., screening, eligibility, included in systematic review, and, if applicable, included in the meta-analysis).

Risk of bias across studies	15	Specify any assessment of risk of bias that may affect the cumulative evidence (e.g., publication bias, selective reporting within studies).
Additional analyses	16	Describe methods of additional analyses (e.g., sensitivity or subgroup analyses, meta-regression), if done, indicating which were pre-specified.
**RESULTS**		
Study selection	17	Give numbers of studies screened, assessed for eligibility, and included in the review, with reasons for exclusions at each stage, ideally with a flow diagram.
Study characteristics	18	For each study, present characteristics for which data were extracted (e.g., study size, PICOS, follow-up period) and provide the citations.
Risk of bias within studies	19	Present data on risk of bias of each study and, if available, any outcome level assessment (see item 12).
Results of individual studies	20	For all outcomes considered (benefits or harms), present, for each study: (a) simple summary data for each intervention group (b) effect estimates and confidence intervals, ideally with a forest plot.
Synthesis of results	21	Present results of each meta-analysis done, including confidence intervals and measures of consistency.
Risk of bias across studies	22	Present results of any assessment of risk of bias across studies (see Item 15).
Additional analysis	23	Give results of additional analyses, if done (e.g., sensitivity or subgroup analyses, meta-regression [see Item 16]).
**DISCUSSION**		
Summary of evidence	24	Summarize the main findings including the strength of evidence for each main outcome; consider their relevance to key groups (e.g., healthcare providers, users, and policy makers).
Limitations	25	Discuss limitations at study and outcome level (e.g., risk of bias), and at review-level (e.g., incomplete retrieval of identified research, reporting bias).
Conclusions	26	Provide a general interpretation of the results in the context of other evidence, and implications for future research.
**FUNDING**		
Funding	27	Describe sources of funding for the systematic review and other support (e.g., supply of data); role of funders for the systematic review.

Data collection process	10	Describe method of data extraction from reports (e.g., piloted forms, independently, in duplicate) and any processes for obtaining and confirming data from investigators.
Data items	11	List and define all variables for which data were sought (e.g., PICOS, funding sources) and any assumptions and simplifications made.
Risk of bias in individual studies	12	Describe methods used for assessing risk of bias of individual studies (including specification of whether this was done at the study or outcome level), and how this information is to be used in any data synthesis.
Summary measures	13	State the principal summary measures (e.g., risk ratio, difference in means).
Synthesis of results	14	Describe the methods of handling data and combining results of studies, if done, including measures of consistency (e.g., I^2) for each meta-analysis.
